# A Mixed-Methods Study of Factors Influencing Access to and Use of Micronutrient Powders in Rwanda

**DOI:** 10.9745/GHSP-D-20-00422

**Published:** 2021-06-30

**Authors:** Theogene Dusingizimana, Janet L. Weber, Thiagarajah Ramilan, Per Ole Iversen, Louise Brough

**Affiliations:** a School of Food and Advanced Technology, Massey University, Palmerston North, New Zealand.; b Department of Food Science and Technology, College of Agriculture, Animal Sciences and Veterinary Medicine, University of Rwanda, Musanze, Rwanda.; c School of Agriculture and Environment, Massey University, Palmerston North, New Zealand.; d Department of Nutrition, Institute of Basic Medical Sciences, University of Oslo, Oslo, Norway.; e Department of Hematology, Oslo University Hospital, Oslo, Norway.; f Division of Human Nutrition, Faculty of Medical Health Sciences, Stellebosh University, Tygerberg, South Africa.

## Abstract

The World Health Organization recommends point-of-use fortification with multiple micronutrients powder (MNP) for foods consumed by children aged 6–23 months in populations where anemia prevalence among children under 2 years or under 5 years of age is 20% or higher. In Rwanda, anemia affects 37% of children under 5 years. The MNP program was implemented to address anemia, but research on factors affecting the implementation of the MNP program is limited. We conducted a mixed-methods study to examine the factors influencing access to and use of MNP among mothers (N=379) in Rutsiro district, northwest Rwanda. Inductive content analysis was used for qualitative data. Logistic regression analysis was used to determine factors associated with the use of MNP. Qualitative results indicated that the unavailability of MNP supplies and distribution issues were major barriers to accessing MNP. Factors influencing the use of MNP included mothers' perceptions of side effects and health benefits of MNP, as well as inappropriate complementary feeding practices. Mothers of older children (aged 12–23 months) were more likely to use MNP than those of younger children (aged 6–11 months) (adjusted odds ratio [aOR]=3.63, *P*<.001). Mothers whose children participated in the supplementary food program were nearly 3 times more likely to use MNP than those whose children had never participated in the program (aOR=2.84, *P*=.001). Increasing household hunger score was significantly associated with lower odds of using MNP (aOR=0.80, *P*=.038). Mechanisms to monitor MNP supply and program implementation need to be strengthened to ensure mothers have access to the product. MNP program implementers should address gaps in complementary feeding practices and ensure mothers have access to adequate complementary foods.

## Abstract (precis)

Gaps in complementary feeding practices hinder the use of multiple micronutrients powder (MNP) in Rutsiro district in Rwanda. Successful MNP program implementation requires uninterrupted availability and accessibility to the product, as well as greater understanding of health benefits of the MNP.

## Teaser

**Key Findings:**

- In Rutsiro district, the unavailability of multiple micronutrients powder (MNP) was mentioned as a major barrier to accessing it. Factors that appeared to limit the use of MNP included perceived side effects and the perceptions that MNP is designed for undernourished children, coupled with inappropriate child feeding practices, particularly feeding thin/watery complementary foods.
- Mothers of older children (aged 12–23 months) had about 4 times higher odds of using MNP than mothers of younger children (aged 6–11 months), while mothers whose children participated in the supplementary food program had about 3 times higher odds of using MNP than mothers whose children have never participated in the program. Food insecurity, as indicated by increasing household hunger score, was significantly associated with reduced odds of using MNP.

- Ensure uninterrupted MNP supply and make MNP available to mothers while enhancing their understanding of the health benefits of MNP.
- Address gaps in complementary feeding practices by emphasizing timely introduction of diverse complementary foods of adequate consistency and supporting mothers' access to adequate complementary foods.

Résumé en français à la fin de l'article.

## INTRODUCTION

Inadequate intake of micronutrients is recognized as one of the most important contributors to the global burden of diseases.^1^ An estimated 2 million children worldwide die (19% of total child deaths) each year due to insufficient intake of micronutrients, mainly iron, vitamin A, and zinc.^1^ Iron deficiency is the most common micronutrient deficiency worldwide,^2^ and it has numerous functional consequences on child health, including impaired physical growth and poor neurocognitive development.^3^^,^^4^

In Rwanda, the prevalence of anemia among children under 5 years of age declined significantly from 52% in 2005 to 38% in 2010,^5^ but the most recent Demographic and Health Survey (DHS) found that 37% of children under 5 years of age had anemia in 2019–2020.^6^ The same survey showed that children aged 6–23 months were the most affected. For example, anemia affects 70% of children aged 6–8 months and 64% of those aged 9–11 months.^6^ Although other factors, such as parasite infections, may contribute to the high rates of anemia, evidence suggests that iron deficiency, resulting from inadequate dietary iron intake and/or low bioavailability and increased needs for iron during child growth, is a major cause.^7^ As in many other low- and middle-income countries, Rwandan children consume predominantly plant-based diets, which contain low bioavailable iron.^8^ A recent study conducted in Rwanda found that >60% of children aged 6–23 months do not meet their requirements for iron and other minerals such as calcium and zinc due to low nutrient density for these micronutrients in complementary foods.^9^ The consumption of iron-rich foods, such as animal-source foods, and commercial fortified infant foods is low among children aged 6–23 months (20% and 2%, respectively).^5^ As a consequence, it's difficult for young children to meet their requirements for iron and other micronutrients during the critical development stages.^10^

The Rwandan food and nutrition policy^11^ recognizes the severity of anemia among Rwandan children and has proposed several solutions, including dietary diversity promotion, food fortification, point-of-use fortification with micronutrient powders, use of biofortified crops (e.g., high-iron beans), and deworming.^11^ The Rwandan government also implements a supplementary food program that aims to address undernutrition in the child's first 1,000 days of life. The program provides fortified complementary blended porridge flour (locally known as *Shisha Kibondo*) to pregnant and lactating mothers as well as young children aged 6−23 months from the most vulnerable households.^12^ Moreover, in 2011, the government of Rwanda, with support from the United Nations Children's Fund (UNICEF), introduced a point-of-use fortification program using multiple micronutrients powder (MNP) as a measure to improve the nutritional quality of complementary foods consumed by children aged 6–23 months and to prevent micronutrient deficiencies among these children.^13^

The point-of-use fortification of complementary foods with iron-containing MNP is recommended when anemia prevalence among young children is 20% or more.^14^ Studies conducted in many countries, including Rwanda, with high burden of anemia demonstrated efficacy of MNP in reducing the prevalence of anemia and iron deficiency among children aged 6–24 months.^15^^,^^16^ While MNP interventions have been shown to be efficacious in many studies, they are often conducted in controlled trials using resources that are not usually available during a national implementation or scale-up.^17^ In some settings, MNP programs have thus been ineffective due, in part, to factors that may affect actual implementation.^18^^,^^19^ For example, a study in Uganda^19^ found that mothers cooked foods with soda ash to reduce cooking time. The authors argued that the ash might have negatively influenced the bioavailability and absorption of micronutrients, making the MNP program ineffective. In addition, contextual factors such as beliefs, resource constraints, and so forth can have an influence on the coverage and utilization of nutrition programs targeting infants and young children.^20^ A review^21^ of 11 studies on coverage of nutrition programs in 5 countries, including MNP programs, reported significant variability in message coverage (i.e., whether respondents have ever heard of the product), contact coverage (i.e., whether the product has ever been fed to the child), or effective coverage (i.e., whether the product has been utilized as per the pre-established program recommended frequency and quantity) due to different real-world delivery/implementation conditions in which the programs were implemented. The review concluded that achieving impact at scale of such programs requires a better understanding of the factors affecting coverage and utilization.^21^ The need for research to understand the factors influencing MNP program implementation in a variety of contexts has been recognized.^22^^,^^23^

The point-of-use fortification of complementary foods with iron-containing MNP is recommended when anemia prevalence among young children is 20% or more.

The purpose of the present study was to examine the factors influencing access to and use of MNP among mothers in Rwanda. In the context of the current study, the MNP program is of interest because the prevalence of anemia has barely changed between 2010 and 2020 in a group of children aged 6–23 months with the highest anemia prevalence,^6^^,^^11^ despite the MNP program being introduced in Rwanda in 2011, adopted by the 2013 national food and nutrition strategic action plan to address anemia in children aged 6–23 months,^11^ and scaled up in all 30 districts of Rwanda in 2017.^13^

The purpose of the present study was to examine the factors influencing access to and use of MNP among mothers in Rwanda.

## METHODS

This study was conducted in Rutsiro district, northwest Rwanda, approximately 140 km from the capital city, Kigali. The district has the highest prevalence of child stunting (54%) among children under 5 years.^24^ The majority (∼98%) of the district's population is rural, and agriculture on small plots of land is the main livelihood.^25^ The main subsistence crops are maize, beans, banana plantain, cassava, and sweet and Irish potatoes. The health system in the district consists of 1 hospital and 17 health centers.^26^ Each health center oversees community health workers (CHWs) who provide community-based nutrition and other health services to an average of 23,000 inhabitants living within the health center's catchment area.^27^

The services provided by CHWs include distribution of MNP, locally known as *Ongera*, to caregivers with children aged 6–23 months. In Rwanda, the Ministry of Health or UNICEF deliver MNP to district hospitals, which then distribute MNP supplies to health centers. MNP is then distributed by the health centers to CHWs, who in turn distribute MNP to caregivers during monthly child growth monitoring and promotion activities. Some nongovernmental organizations, mainly World Vision International (Rwanda) and Caritas Rwanda, support the MNP program implementation through training of CHWs and awareness activities related to child feeding. Every caregiver with a child aged 6−23 months is entitled to 30 sachets of MNP per month, which they receive free of charge. Using cooking demonstrations, CHWs also counsel caregivers on optimal complementary feeding practices, such as age-specific dietary diversity, consistency and quantity of complementary foods, and on MNP usage.^8^^,^^13^

Every caregiver with a child aged 6−23 months is entitled to 30 sachets of MNP per month, which they receive free of charge.

### Study Design and Participants

This study used a cross-sectional convergent mixed-methods design,^28^ combining both quantitative and qualitative data. The data used in this study were collected as part of a survey conducted between September 2018 and January 2019 to investigate the factors associated with nutritional status of children aged 6–23 months. Details on the survey sample size estimation and participants recruitment are described elsewhere.^29^ Briefly, the district was first divided into 3 zones based on main roads connecting the district to its neighboring districts. In each zone, 3 health centers were purposely selected to maximize geographic distribution, for a total of 9 health centers. Within each of the selected health center's catchment area, 2 villages were randomly selected. In these villages, monthly growth monitoring lists were obtained from CHWs and used to compile a sampling frame from which participants were randomly selected. Mothers who refused to participate and those who were not found in their homes were replaced (11 mothers in total) by selecting the next name on the list. Eligibility criteria were (1) having a child aged 6–23 months; (2) child was apparently healthy (i.e., no overt signs of illness); and (3) being in the 2 lowest socioeconomic categories. Of the 400 survey participants, 21 (5%) of the children were excluded from the analysis due to premature birth (i.e., before 37 weeks of gestation) or low birthweight (i.e., less than 2.5 kg). The remaining 379 participants formed the basis of the present study.

### Data Collection

Quantitative and qualitative data were collected concurrently using a survey questionnaire. The questionnaire was developed in English, translated into Kinyarwanda, and programmed into a handheld tablet (Samsung Galaxy Tab 8.0 T295, Korea). It was pretested, and data were collected through face-to-face interviews. Qualitative data were audio-recorded.

### Ethics

This study was approved by the Massey University Human Ethics Committee (reference: SOA 17/67) and the Institutional Review Board of the University of Rwanda's College of Medicine and Health Sciences (reference: 003/CMHS IRB/2017). Permission to collect data was also obtained from the Rutsiro District Public Health Office. Oral informed consent was obtained from all participants.

### Quantitative Data

#### Outcome Variable

“Ever using MNP” was the primary outcome variable. Mothers were asked if they added (yes/no) MNP to the target child's foods in the last 7 days prior to the survey. Mothers who had not used MNP were asked whether they had ever used MNP before (yes/no). A mother was categorized as “ever used MNP” if she had used MNP in the previous 7 days or before, and those who had not used MNP either within 7 days prior to the survey or before were categorized as “never used MNP.”

#### Other Variables

Information related to participants' demographics, socioeconomic, household food security, and indicators of health system engagement were obtained through mothers' recall. Health cards were used for verification (e.g., child age and heath information). Demographic information reported by mothers included the child's age and sex and the maternal age at first birth. Mothers also reported presence of symptoms of child diarrhea (defined as ≥3 watery or loose stools per day) and upper respiratory infections (runny nose, coughing, or wheezing) in the previous 4 weeks. Socioeconomic variables included maternal education level (coded as none/incomplete primary education, complete primary education, secondary education) and household asset ownership (e.g., radio, land, domestic animals, housing characteristics). Fourteen household assets were used to create a household wealth index using principal component analysis.^30^ The first component was taken to represent the household wealth index and divided into terciles (lower, middle, and upper). A household hunger score—a proxy of a household's ability to access food—was measured using a validated cross-cultural household hunger scale (HHS).^31^ Adhering to HHS measurement guide, mothers were asked 3 questions intended to capture 3 situations (i.e., lack of food of any kind in the house; going to sleep hungry because there was not enough food; and going a whole day and night without eating) reflecting a household's experience of insufficiency of food supply and intake and physical consequences. Each question was followed by the frequency-of-occurrence question (i.e., how often the reported situation was experienced). The responses were coded and used to generate a household hunger score that ranged from 0 (indicating no hunger) to 6 (indicating severe hunger). Indicators of health system engagement are (1) attendance at growth monitoring site in the previous month (coded as yes/no); (2) the number of antenatal care visits when pregnant with the study child (coded as <4 visits or ≥4 visits; a minimum of 4 visits is recommended in Rwanda^5^); and (3) whether the child ever participated in the supplementary food program (coded as yes/no).

A household hunger score—a proxy of a household's ability to access food—was measured using a validated cross-cultural household hunger scale.

### Qualitative Data

The questionnaire included an open-ended question that was used to collect in-depth information on the reasons for not using MNP. Mothers who had not used MNP in the previous 7 days (i.e., those who used MNP but not in the previous 7 days, and those who never used MNP) were asked to provide reasons for not using MNP. Probes (either open-ended or specific to the mothers' comments) were used to obtain additional information.^32^

### Data Analysis

#### Quantitative Data

Median (interquartile range [IQR]) values were determined for continuous data and percentages for categorical data. Bivariate and multiple logistic regression analyses were performed to examine factors associated with using MNP. The full model adjusted for the presence of diarrhea and respiratory infection in the past 4 weeks. We adjusted for these variables because our previous research in the same population showed that child illness has negative effects on how mothers feed their children, including withholding or restricting some foods from children's diets.^33^ Unadjusted and adjusted odds ratios (OR) and 95% confidence intervals (CI) were computed. Variables with a *P* value of <.05 were considered significant predictors. We did not perform a Bonferroni correction because, although the correction decreases the probability for type I error, such adjustment is vulnerable to type II error and can obscure important findings.^34^^,^^35^ All statistical analyses were performed using SPSS version 25.0 (IBM Corp., Armonk, NY).

#### Qualitative Data

Mothers' responses were audio-recorded, transcribed verbatim in Kinyarwanda, and translated into English. Content analysis^36^ was used to analyze the data. An inductive content analysis approach, which is recommended when there is no prior research or little is known about the studied phenomenon, was used. The data analysis had 3 phases: preparation, organization, and reporting.^37^ The first phase consisted of careful reading of the data several times to become immersed in and familiar with the data. In the organization phase, each transcript was read carefully by the first author, highlighting the text (words or phrases) that appeared to describe the phenomenon under study (i.e., access to and/or use of MNP). The highlighted texts were openly and manually coded by giving each text a descriptive code. The second author read the data to confirm the descriptive codes. These codes were revised, and the codes that emerged from the revision were jointly reviewed before integrating them into the analysis. Final codes were examined, compared, and grouped into categories that represented similar meaning.^38^ The first, second, and last authors reviewed, discussed, and agreed on the final code categories. In the final phase of analysis, SPSS (version 25) was used to quantify the frequency of major categories and subcategories.^39^ To interpret and report the findings, examples of original textual responses representing specific code or category are presented.

## RESULTS

### Quantitative Results

Characteristics of the participants (N=379) are presented in Table 1. The median (IQR) age of children was 15 (11–19) months, whereas the median (IQR) age of mothers at first birth was 22 (20–24) years. More than a half (59%) of the mothers had either no education or did not complete primary education, and only 35% of the mothers had ≥4 antenatal care visits during their last pregnancy. The median (IQR) household size was 4 (3–6) members.

**TABLE 1.** Sociodemographic and Nutritional Characteristics of Participants (N=379) in a Study on Access to and Use of MNP in Rutsiro District, Rwanda, September 2018–January 2019

Characteristic	
Child age, months, median (IQR)	15 (11–19)
Child age group, No. (%)	
6–11 months	120 (32)
12–23 months	259 (68)
Sex, No. (%)	
Male	184 (49)
Female	195 (51)
Child had diarrhea (past 4 weeks), No. (%)	179 (47)
Child had respiratory infection (past 4 weeks), No. (%)	297 (78)
Child ever participated in the supplementary food program, No. (%)	283 (75)
Mother's age at first birth, years, median (IQR)	22 (20–24)
Mother's education level,^a^ No. (%)	
Illiterate/incomplete primary	219 (59)
Completed primary	88 (24)
Some secondary	62 (17)
No. of antenatal care visits attended, No. (%)	
1–3	248 (65)
≥4	131 (35)
Mother attended child growth monitoring site (past month), No. (%)	318 (84)
Household hunger score, median, (IQR)	1 (1–2)
Household size, median (IQR)	4 (3–6)
Wealth index terciles, No. (%)	
Lower	126 (33)
Middle	126 (33)
Upper	127 (34)

Abbreviations: IQR, interquartile range; MNP, multiple micronutrients powder.

a Owing to missing data, n=369.

The majority of the mothers (64%) reported ever adding MNP to their children's food, but only 38% added it to their children's food in the previous 7 days (Table 2). The proportion of mothers using MNP to feed their children was significantly lower among mothers with children aged 6–11 months than among those with children aged 12–23 months (39% vs. 76%, *P*<.001) (results not shown).

**TABLE 2.** Proportion of Mothers Who Used/Did Not Use MNP to Feed Their Children (N=379), by Age Group, in Rutsiro District, Rwanda, September 2018–January 2019

Age Group, Months	No.	Used MNP in the Past 7 Days, No. (%)	Used MNP but Not in the Past 7 Days, No. (%)	Ever Used MNP,^a^ No. (%)	Never Used MNP, No. (%)
6–8	57	10 (17)	2 (4)	12 (21)	45 (79)
9–11	63	22 (35)	13 (21)	35 (56)	28 (44)
12–23	259	113 (44)	83 (32)	196 (76)	63 (24)
Total	379	145 (38)	98 (26)	243 (64)	136 (36)

Abbreviation: MNP, multiple micronutrients powder.

a Sum of “used MNP in the past 7 days” and “used MNP but not in the past 7 days.”

The majority of the mothers (64%) reported ever adding MNP to their children's food, but only 38% used it in the previous 7 days.

Table 3 shows that mothers of older children (aged 12–23 months) had about 4 times higher odds of using MNP than those of younger children (aged 6–11 months) (aOR=3.63, *P*<.001). Similarly, mothers whose children ever participated in the supplementary food program had about 3 times higher odds of using MNP than the mothers whose children have never participated in the program (aOR=2.84, *P*<.001). Conversely, as the household hunger score increases by 1 unit, the odds of using MNP decreased significantly by about 20% (aOR=0.80, *P*=.038). Even though attendance to growth monitoring in the last month was a significant predictor of the use of MNP, this variable was not significant in the adjusted model (the multivariable model adjusted for the presence of diarrhea and respiratory infections in the past 4 weeks).

**TABLE 3.** Factors Associated With the Use of MNP in Rutsiro District, Rwanda, September 2018–January 2019^a^

Variables	COR (95% CI)	*P*-Value	aOR (95% CI)	*P*-Value
Child age group		<.001		<.001
6–11 months	1		1	
12–23 months	4.83 (3.04, 7.68)		3.63 (2.14, 6.16)	
Child sex		.52		.53
Female	1		1	
Male	0.87 (0.57, 1.33)		0.86 (0.53, 1.39)	
Maternal age at first birth	1.03 (0.96, 1.09)	.41	1.01 (0.94, 1.09)	.82
Maternal education level		.32		.44
None/incomplete primary education	1		1	
Complete primary education	1.12 (0.67, 1.88)		1.01 (0.56, 1.83)	
Some secondary education	1.62 (.87–3.01)		1.57 (0.77, 3.19)	
ANC visits		.82		.60
<4 visits	1		1	
≥4 visits	0.95 (0.61, 1.47)		0.87 (0.53, 1.45)	
Mother attended GM (past month)		.01		.43
No	1		1	
Yes	2.10 (1.21, 3.45)		1.31 (0.68, 2.53)	
Child ever participated in the supplementary food program		<.001		.001
No	1		1	
Yes	4.54 (2.78, 7.41)		2.84 (1.57, 5.13)	
Household hunger score	0.89 (0.76, 1.05)	.18	0.80 (0.65, 0.99)	.038
Household wealth index		.77		.26
Lowest tertile	1		1	
Middle tertile	1.20 (0.71, 2.00)		0.83 (0.45, 1.56)	
Upper tertile	1.01 (0.61, 1.69)		0.59 (0.31, 1.13)	

Abbreviations: ANC, antenatal care; aOR, adjusted odds ratio; CI, confidence interval; COR, crude odds ratio; GM, growth monitoring; MNP, multiple micronutrients powder.

a The multivariable model was adjusted for the presence of child diarrhea and respiratory infection in the previous 4 weeks.

### Qualitative Results

Factors influencing access to and use of MNP are summarized into 9 categories. Below we present results for 6 major categories. A summary of these 6 categories, as well as other 3 minor categories are presented in a Supplement.

#### Limited Availability of MNP Supplies

A frequently reported barrier to accessing MNP was the lack of supplies. Many mothers (n=72) reported getting information from CHWs that there was no MNP stock at their health centers. For example, one mother said:

*I don't have Ongera (MNP) now. We get it from health workers but this month they said they don't have Ongera in the stock at the health center. They told us to come on 13th of November when we take children for growth monitoring*.

A frequently reported barrier to accessing MNP was the lack of supplies.

#### CHWs–Mother Interactions

Most mothers reported receiving MNP from CHWs through monthly growth monitoring activities while others reported occasionally receiving MNP from health centers (e.g., when mothers took children there for immunization). However, information received from CHWs in the study area indicated that, if mothers ran out of MNP prior to the next distribution date, the mothers were encouraged to contact CHWs and acquire more MNP sachets, if available. However, some mothers (n=33) said that they preferred to wait for routine distribution of MNP, which they felt was the responsibility of CHWs:

*I don't have Ongera [MNP]. We used all the sachets that we had received at the village kitchen. I am waiting for our CHW to distribute Ongera. I don't go to her house to ask for Ongera because they [CHWs] are supposed to distribute. Many times, we were told: wait, wait. So, I prefer to wait, and if I don't have Ongera, it's their fault.*

The narratives also suggested that interactions between mothers and CHWs may be limited, by nonattendance to growth monitoring sites due to competing obligations (n=6), such as the need to work for income to meet family needs. One mother said:

*I received Ongera once; we used them up all. It's been a while without attending growth monitoring session. Most of the days for growth monitoring are days that I am working. Working is most important. It's how we get money to survive.*

#### Limited Information

Some mothers (n=16) explicitly said that they did not know about MNP. A few mothers reported having heard about but not seen MNP (n=4); others mentioned that they were unaware of the distribution schedules or eligibility criteria (n=6). One mother said:

*I have never received Ongera. I think there are some children who are eligible … may be those that can feed themselves …, and others who are not eligible.*

#### Perceived Side Effects

Some mothers (n=19) reported that they stopped using MNP to feed their children because of side effects experienced by their children after consumption of MNP. Diarrhea was the most frequently reported side effect experienced, while other side effects included vomiting and fever. While most mothers who reported side effects spoke from their own children's experiences, a few mothers decided not to use MNP because of the comments by other mothers in their community about the negative side effects of MNP:

*I received Ongera this month when he just turned 9 months. I fed him Ongera, like 3 times, and then he started having diarrhea. So, I stopped adding it to his foods.*

*I received 30 sachets of Ongera last month. I just kept them. I heard from other mothers that Ongera causes diarrhea, so I never fed it to my child.*

Some mothers reported that they stopped using MNP to feed their children because it caused side effects or made food unpalatable.

Several mothers (n=20) also linked MNP with changes in taste of food, stating that their children disliked foods that were prepared with MNP. One mother said:

*We have used all the sachets [of Ongera] that we received this month. But even when it was still available, he did not really like the foods when it was mixed with Ongera. I used to give him and skipped some days.*

#### Incompatibility Between MNP Program Recommendations and Current Child-Feeding Practices

Several mothers (n=33), especially those with younger children (aged 6–8 months), reported never using MNP due to reasons related to feeding practices. For example, many of these mothers (n=27) said that their “child had just started eating” complementary foods or that the “child was still adapting to complementary foods.” Others (n=6) mentioned that they had not yet introduced complementary foods to the child:

*I obtained Ongera a week ago, but I have not yet started giving it to my child. She is not yet ready for all foods. She is only 7 months. We give her things like a spoon of porridge or soft Irish potatoes. I mash them with my fingers. We don't add Ongera in the porridge. We add sugar, not Ongera.*

#### MNP Was Perceived as Intended for Malnourished Children

Some mothers (n=11) perceived that MNP is intended for malnourished children, and children who appeared healthy did not require MNP. When asked to justify their judgment regarding the healthiness of their children, the mothers explained that their child was visually not sick or did not show any signs of malnutrition. Physical appearance, weight loss, and hair discoloration were the most common signs used to describe healthiness of the children:

*I know I can get Ongera from our village health worker. I don't have them now because I never asked. These [Ongera] are intended for malnourished children. My child has no health issues. He is healthy*.

*I don't have Ongera [MNP]. I have never fed him Ongera. Ongera are given to children who have bwaki (local term used to denote acute malnutrition). You don't know bwaki? It is when your child has golden hair and swollen tummy.*

Some mothers perceived that MNP is intended for malnourished children, and children who appeared healthy did not require MNP.

## DISCUSSION

Limited information is available on the factors influencing access to and use of MNP in Rwanda. In the present study, we found that the proportion of mothers who added MNP to their children's foods in the previous 7 days was low (38%), especially those with younger children aged 6–11 months. This proportion increased to 64% when considering all mothers who reported ever having added MNP to their children's foods. In a small-scale survey of 186 caregivers recruited from 19 of Rwanda's 30 districts that implemented MNP program (n=10 caregivers in each district), McLean et al.^13^ reported 87% coverage (defined as the proportion of caregivers receiving a box of MNP in the previous 3 months). It is worth mentioning that our study found that some mothers who received MNP still did not feed it to their children, so while receiving MNP is sometimes used as an indicator of program coverage, whether the child is consuming MNP according to the program recommended quantity and frequency, and not simply receiving it, may be a better indicator of program success.^21^ No consensus exists on a cutoff value for satisfactory MNP program coverage; however, one suggestion is that effective MNP program performance should be appraised as satisfactory when >70% of target children are found (at the time of study) to be consuming MNP.^40^ Although estimating actual coverage was beyond the scope of this study, our results suggest that the MNP program coverage in Rutsiro district is generally low. Further research to assess MNP coverage in Rwanda using appropriate frameworks is recommended. For example, Tanahashi's framework^41^ has been widely used to assess health service or intervention coverage and to identify implementation bottlenecks. This framework defines different stages of coverage, including availability, accessibility, acceptability, contact, and effectiveness. Availability coverage refers to the availability of resources (e.g., drugs, health workers, health facilities) that determine the extent to which an intervention can be made available to the target population. Accessibility coverage is the proportion of the target population for whom an intervention is accessible. Acceptability coverage is the number of people who are willing to use an accessible intervention (they must find it acceptable in terms of, for example, cost, waiting time, beliefs). Contact coverage is the number of people who have been in contact with an intervention and have used it. Effectiveness coverage is the proportion of the target population in need of an intervention that receives an effective intervention.^41^

In this study, a majority of mothers mentioned lack of MNP supplies as the major issue limiting their access to MNP. Limited availability of MNP supplies has been identified in this study, as in others, as a major constraint to access to MNP in many countries.^42^^,^^43^ In Rwanda, lack of supplies and inadequate distribution of MNP were also reported as key obstacles limiting MNP program coverage.^13^ These findings highlight the need to ensure uninterrupted MNP supply to increase coverage of the MNP program. However, even when MNP is available, there are factors related to MNP distribution arrangements that need to be considered. For example, while mothers were encouraged to pick up MNP sachets from their village CHWs, our data indicated that the mothers' expectation was to obtain MNP through routine distribution; however, some narratives suggested there may be opportunity costs associated with attending the distribution sites. This finding implies that the health system must ensure that mothers obtain MNP at no extra time cost. Also, instead of a monthly MNP supply, it may be useful to provide mothers with quantities that are sufficient for several months. If the aim is for mothers to actively seek out MNP, it is essential that they understand its health benefits.

Limited availability of MNP supplies has been identified in this study, as well as others, as a major constraint to access to MNP.

Consistent with other studies,^17^^,^^42^^,^^44^^,^^45^ qualitative results from the present study showed that perceived side effects (e.g., diarrhea, vomiting) and change in taste of foods mixed with MNP were among barriers to using MNP. It has been suggested that possible changes to foods due to addition of MNP and the potential negative side effects of MNP should be acknowledged and clearly communicated to caregivers before children start getting MNP.^23^ Moreover, our results revealed that some mothers hold the belief that MNP is mainly for undernourished children. According to these mothers, giving MNP to their (perceived) healthy children was unnecessary. It is important to highlight that micronutrient deficiencies (also known as hidden hunger) such as anemia often have no visible or immediate signs and can coexist with other forms of undernutrition such as stunting, which is also not easily recognized.^46^^,^^47^ Therefore, the belief among mothers that children do not need MNP because they lack overt symptoms of ill health or undernutrition requires further attention because it presents important challenges for mothers, health professionals, as well as for MNP program implementers. On one hand, such beliefs may undermine the demand for and use of MNP among mothers. On the other hand, the beliefs may make it difficult for the health professionals and MNP program implementers to raise awareness among mothers about MNP. Research shows that belief is a key determinant of maternal health care seeking behavior.^48^ For example, a study conducted in Kenya found that parents who considered MNP as a drug were reluctant to use it in the absence of explicit child illness.^49^ These findings point to the need for appropriate health messages to ensure mothers understand the health benefits and need for MNP intervention. More specifically, clear and straightforward messages such as “children can still suffer from micronutrient deficiencies even when they are visually healthy” must be used. However, simple words that are adapted to the setting and mothers' level of education should be used to describe micronutrient deficiencies for a better understanding. It has been suggested that, unless there is some perceived need, individuals may not use an intervention, even if it is free.^50^

The belief among mothers that children do not need MNP because they lack overt symptoms of ill health or undernutrition requires further attention.

MNP programs are designed such that children should start receiving MNP as soon as they are aged 6 months old. In the current study, we found that mothers of younger children were less likely to use MNP than mothers of older children. Similar findings have been reported in Nepal,^51^ where MNP program coverage was lower among younger children (aged 6–11 months) than among older children (aged ≥12 months). In Mongolia, it was also reported that parents delayed feeding MNP to their children until an average age of 13 months.^52^ The authors of these 2 studies did not elucidate the factors responsible for the delay in feeding MNP to young children. Qualitative results from the present study suggested that the delay in receiving MNP by younger children was due, in part, to the current complementary feeding practices. For example, our data indicated that half of the mothers with children aged 6–8 months reported that they had never used MNP, either because their child “was still adapting to complementary foods” or because the child “had not been introduced to complementary foods.” Previous studies conducted in Rwanda showed that children were introduced to complementary foods later than recommended (i.e., aged 8 months),^13^ and that dilute cereal porridges were the main food given to young children who were aged 2–8 months.^53^ A recent study^33^ conducted in the same population also found that thin porridges and stews/soups were the most common foods given to young children, and that the consistency of these foods hindered the use of MNP. In addition, MNP must be mixed with thick solid or semisolid complementary foods because it dissolves in liquids, which may change the taste or color of the foods, leading to less acceptance by children.^54^ However, the recommendation to mix MNP with thick/solid or semisolid foods that are introduced to children at a later stage is likely to delay the introduction of MNP to younger children. Therefore, rather than discouraging mothers from using MNP with porridges or other soft foods, an alternative approach is to teach mothers how to improve the consistency of these foods by using local ingredients such as ground nut or bean flours. Once an improved porridge/stew is accepted and feasibility to use it as a vehicle for MNP explored, it could facilitate the mothers feeding MNP to children using a culturally accepted and age-appropriate food vehicle. A similar approach has been found to be successful in Mali.^55^

Our findings also showed that access to food is a predictor of using MNP. We found that the odds of using MNP reduced significantly with increasing household hunger score. Although not necessarily a direct cause, this may be a marker of other factors related to poverty that may play a causal role in access or use of MNP. Another study in Niger found that mothers were unable to give MNP to their children simply because they lacked foods to mix with MNP.^56^ Results from the present study also showed that being a beneficiary of the supplementary food program (*Shisha Kibondo*) was associated with higher odds of using MNP. However, it is worth noting that the supplementary food program distributes a fortified cereal-based flour used to prepare porridge, which is not recommended for mixing with MNP. Thus, the influence of the supplementary food program on the use of MNP needs further exploration. Nevertheless, Rutsiro remains the most food insecure district in Rwanda, with 62% of households consuming an inadequate diet in 2018.^24^ In the context of such a widespread food insecurity, mothers' ability to appropriately use MNP may be limited. Therefore, addressing anemia through the MNP program will require, in addition to ensuring availability of and appropriate use of MNP, improvements in the household access to adequate foods.

Our findings also showed that access to food is a predictor of using MNP.

### Strengths and Limitations

The strength of the current study is the integration of both quantitative and qualitative approaches. Limitations of this study include a cross-sectional design, which only demonstrates association and not causal relationships. Another important limitation is that the study looked at a program performance in terms of ever-use and use within the past 7 days. In addition, the study focused on 1 district, and the sample was drawn from purposefully selected health centers. Thus, the findings may not be generalizable to the studied district or to other districts. Moreover, our qualitative findings are based on mothers' perspectives, but it would be important to understand the perspectives of other key informants such as CHWs and health center managers on how to improve uptake and use of MNP. This line of investigation could potentially provide additional insights into other context-specific factors that may also inform the MNP program implementation.

## CONCLUSIONS

Findings from the current study point to several issues that need to be addressed to improve the MNP program implementation in Rwanda. The findings suggest the need for more robust supply-chain management to gauge the continuity of MNP supply and availability at the community level. However, even if MNP supply issues are addressed, it remains crucial to address gaps in complementary feeding practices, including inappropriate consistency of complementary foods and maternal perceptions about young children's developmental ability to consume a variety of foods, while enhancing mothers' access to foods. In order to increase the demand and use of MNP, the program implementers must also ensure that mothers have a clear understanding of the health benefits of MNP. Lastly, future research should examine the effect of other factors, including the quality of information and the frequency of interactions between CHWs and mothers on MNP program implementation.

## Supplementary Material

20-00422-Dusingizimana-Supplement.pdf

## Translation

**En français**

**Une étude par méthodes mixtes des facteurs qui influencent l'accès et l'utilisation des poudres de micronutriments au Rwanda.**

**MESSAGE CLÉ:** Les lacunes dans les pratiques d'alimentation complémentaire entravent l'utilisation des poudres de micronutriments multiples (PMN) dans le district de Rutsiro au Rwanda. La réussite de la mise en œuvre du programme de PMN nécessite une disponibilité et une accessibilité ininterrompues du produit, ainsi qu'une meilleure compréhension des avantages du PMN pour la santé.

**RÉSUMÉN**

L'Organisation Mondiale de la Santé recommande l'enrichissement de l'alimentation à domicile (enrichissement sur le point d'utilisation) à l'aide des poudres de micronutriments multiples (PMN) pour les aliments consommés par les enfants âgés de 6 à 23 mois dans les populations où la prévalence de l'anémie chez les enfants de moins de 2 ans ou 5 ans est de 20% ou plus. Au Rwanda, l'anémie touche 37% des enfants de moins de 5 ans et le programme de PMN a été mis en œuvre pour lutter contre l'anémie. Cependant, la recherche sur les facteurs qui affectent la mise en œuvre du programme de PMN est limitée. Nous avons mené une étude par méthodes mixtes pour examiner les facteurs qui influencent l'accès des mères (n=379) à la PMN et son utilisation dans le district de Rutsiro, au nord-ouest du Rwanda. L'analyse du contenu inductif a été utilisée pour les données qualitatives. Pour déterminer les facteurs associés à l'utilisation des PMN, une régression logistique a été utilisée. Les résultats qualitatifs ont indiqué que l'indisponibilité des approvisionnements en PMN et les problèmes de distribution constituaient des obstacles majeurs à l'accès à la PMN. Les facteurs qui influencent l'utilisation des PMN comprenaient les perceptions, chez les mères, des effets secondaires et des avantages des PMN pour la santé, ainsi que des pratiques d'alimentation complémentaire inappropriées. Les mères d'enfants plus âgés (12 à 23 mois) étaient plus susceptibles d'utiliser la PMN que celles d'enfants plus jeunes (6 à 11 mois) (odds ratio ajusté [ORA]=3,63, *P*<0,001). Les mères des enfants qui avaient participé au programme d'alimentation complémentaire étaient près de 3 fois plus susceptibles d'utiliser la PMN que celles des enfants qui n'avaient jamais participé au programme (ORA=2,84, *P*=0,001). L'augmentation du score de faim dans les ménages était significativement associée à des chances plus faibles d'utiliser la PMN (ORA=0,80, *P*=0,038). Les mécanismes de suivi de l'approvisionnement en PMN et de la mise en œuvre du programme doivent être renforcés pour s'assurer que les mères ont accès au produit. Les responsables de la mise en œuvre du programme de PMN devraient combler les lacunes au niveau des pratiques d'alimentation complémentaire et veiller à ce que les mères aient accès à des aliments complémentaires adéquats.

**Peer Reviewed**

**First published online:** May 24, 2021.
