# Supplementary material for: A Mixed-Methods Study of Factors Influencing Access to and Use of Micronutrient Powders in Rwanda
Source: Glob Health Sci Pract. 2021 Jun 30;9(2):274–85. doi: 10.9745/GHSP-D-20-00422 (PMC8324192; doi:10.9745/GHSP-D-20-00422)
Supplement: 20-00422-Dusingizimana-Supplement.pdf [file 20-00422-Dusingizimana-Supplement.pdf]

Supplement to: Dusingizimana T, Weber JL, Ramilan T, Iversen PO, Brough L. A mixed-methods study of factors influencing access to and use of micronutrient powders in Rwanda. *Glob Health Sci Pract*. 2021;9(2). https://doi.org/10.9745/GHSP-D-20-00422

Supplement Table. Frequency Counts and Percentages of Major and Sub-categories of Factors Influencing Access to and Use of MNP in Rutsiro District, Rwanda, September 2018–January 2019 (n= 234) [1]

	Major categories	Sub-categories	Codes
**Factors related to access to MNP**	Limited availability of MNP supplies<br>74 (32%)	CHWs have no MNP<br>72 (30.7%)	Informed by CHWs about lack of supplies
		CHWs have limited quantity of MNP for all mothers attending GM<br>2 (0.8%)	Mothers arriving late at the GM site have no chance to get MNP
	CHWs–mother interactions<br>41 (17.5%)	MNP routine distribution is more convenient<br>33 (14.1%)	Choice to wait for routine distribution over travelling to CHW's house to get MNP supplies
		Opportunity cost<br>6 (2.5%)	Choice to work for income over attending taking child to GM
		Other<br>2 (1.3%)	Neglect; unfair treatment by CHW
	Limited information about MNP<br>27 (11.5%)	Mothers do not know MNP<br>17 (7.2%)	Mother never heard about MNP<br>Mother heard about but never seen MNP
		Limited information MNP programme<br>10 (4.3%)	Unawareness of eligibility criteria<br>Unawareness of distribution mechanisms
**Factors related to the use of MNP**	Perceived side effects<br>38 (16.2%)	MNP causes child illness<br>19 (8.1%)	MNP causes diarrhoea/vomiting/fever
		Child dislikes foods prepared with MNP<br>20 (8.5%)	MNP changes taste of foods
	Incompatibility between current complementary feeding practices and MNP programme recommendations<br>33 (14.1%)	Child still adapting to CF<br>27 (10.6)	Child is fed thin/watery foods with consistency not recommended by MNP program
		Late introduction to CF<br>6 (2.6%)	Child hasn't started CF
	MNP perceived as intended for malnourished children<br>11 (4.7%)	MNP is meant for malnourished children<br>11 (4.7%)	Healthy children don't need MNP (a child who hasn't lost weight/whose hair looks healthy; has never had health issues)
	MNP is not as important as "real" food	Demotivation due to non-participation in the supplementary program which distribute foods	Children need "real" foods

Supplement to: Dusingizimana T, Weber JL, Ramilan T, Iversen PO, Brough L. A mixed-methods study of factors influencing access to and use of micronutrient powders in Rwanda. *Glob Health Sci Pract*. 2021;9(2). https://doi.org/10.9745/GHSP-D-20-00422

	**Major categories**	**Sub-categories**	**Codes**
	5 (2.1%)	4 (1.7%)	
		No need for MNP if a child eats all foods<br>1 (0.4)	MNP have no additional health benefits
	Lack of recommended foods<br>4 (1.7%)	Economic constraints to access foods<br>2 (0.8%)	Lack of means to buy foods
		Perceived poor quality of available foods<br>2 (0.8%)	Available foods (e.g. sweet potatoes) are not appropriate
	Miscellaneous<br>6 (2.6%)	–	Received expired product<br>MNP left in previous rental house<br>Perceived inability to follow MNP feeding instructions<br>Undisclosed (personal) reasons<br>Child's lack of appetite<br>Child's ill-health

Abbreviations: CF, complementary foods; CHWs, community health workers; MNP, multiple micronutrients powder; GM, growth monitoring.
Note: The frequency was determined by counting the number of times a major/sub-category occurred divided by the number of participants. The frequencies do not add up to 100% as some mothers were coded more than once.
